# Tris(4-chloro­phen­yl) phosphate

**DOI:** 10.1107/S2414314624010617

**Published:** 2024-11-08

**Authors:** Joshua Birch, Eric Cyriel Hosten, Richard Betz

**Affiliations:** a Nelson Mandela University, Summerstrand Campus, Department of Chemistry, University Way, Summerstrand, PO Box 77000, Port Elizabeth, 6031, South Africa; University of Aberdeen, United Kingdom

**Keywords:** crystal structure

## Abstract

The title compound, C_18_H_12_Cl_3_O_4_P, is the symmetric phosphate derived from *para*-chloro­phenol and phospho­ric acid. Two of the three aromatic moieties adopt *syn*-orientation towards the P=O bond while the last chloro­phenol ring is pointing away from this bond. In the extended structure, C—H⋯O bonds connect the individual mol­ecules into sheets lying perpendicular to the crystallographic *b* axis.

## Abstract 2

In the title compound, the symmetric phosphate derived from *para*-chloro­phenol and phospho­ric acid, two of the three aromatic moieties adopt *syn*-orientation towards the P=O bond while the last chloro­phenol ring is pointing away from this bond. In the extended structure, C—H⋯O bonds connect the individual mol­ecules into sheets lying perpendicular to the crystallographic *b* axis.

## Graphical abstract

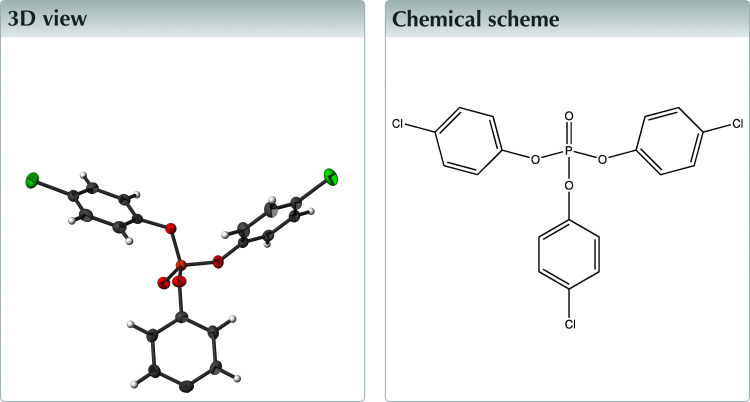

## Structure description

Penta­coordinate compounds of phospho­rus have been an intriguing field of research for many decades, owing to their assumed importance as transition states in biological systems that see the formation and decomposition of a multitude of compounds derived from phospho­ric acid encountered along the many metabolic pathways in eucaryontic cells (Stryer, 1988 ▸; Westheimer, 1968 ▸; Gerlt *et al.*, 1975 ▸; Holmes, 1998 ▸, 2004 ▸). An easy inroad into such derivatives stems from exploiting ligand-exchange reactions starting from already penta­coordinate precursors of this element. At the onset of a study into the chemical reactivity of this class of compound, we sought to synthesize the symmetric oxyphospho­rane of *para*-chloro­phenol, which would result in the formation of a solid reaction product of which a crystalline specimen could be subjected to diffraction studies. The results of the latter showed the presence of a defined hydrolysis product whose formation was already confirmed by means of NMR studies on the crude reaction mixture. In our ongoing inter­est into structural aspects of the heavier pnictogen elements such as phospho­rus (Hosten *et al.*, 2012 ▸; Betz & Klüfers, 2008 ▸; Betz, 2015 ▸; Betz *et al.*, 2011*a* ▸), arsenic (Betz *et al.*, 2007 ▸, 2008 ▸, 2009*a* ▸,*b* ▸, 2011*b* ▸; Betz & Klüfers, 2009 ▸) and anti­mony (Betz *et al.*, 2009*b* ▸, 2010 ▸) and to spare future researchers the waste of valuable data-collection time on diffractometers, the outcome of our crystallographic studies is presented herein. Structural data for the thio­nated analogue of the title compound are apparent in the literature (Hernandez *et al.*, 2006 ▸), as are data for a dinuclear diphos­phazene (Allcock *et al.*, 1994 ▸). Furthermore, the crystal and mol­ecular structures of several ruthenium coordination compounds employing the trivalent phosphite ester as a ligand (Bidal *et al.*, 2019 ▸) as well as two related phospho­ranes in line with the actual intended oxyphospho­rane have been reported (Sarma *et al.*, 1976 ▸; Marczenko *et al.*, 2019 ▸).

**Figure 1:**
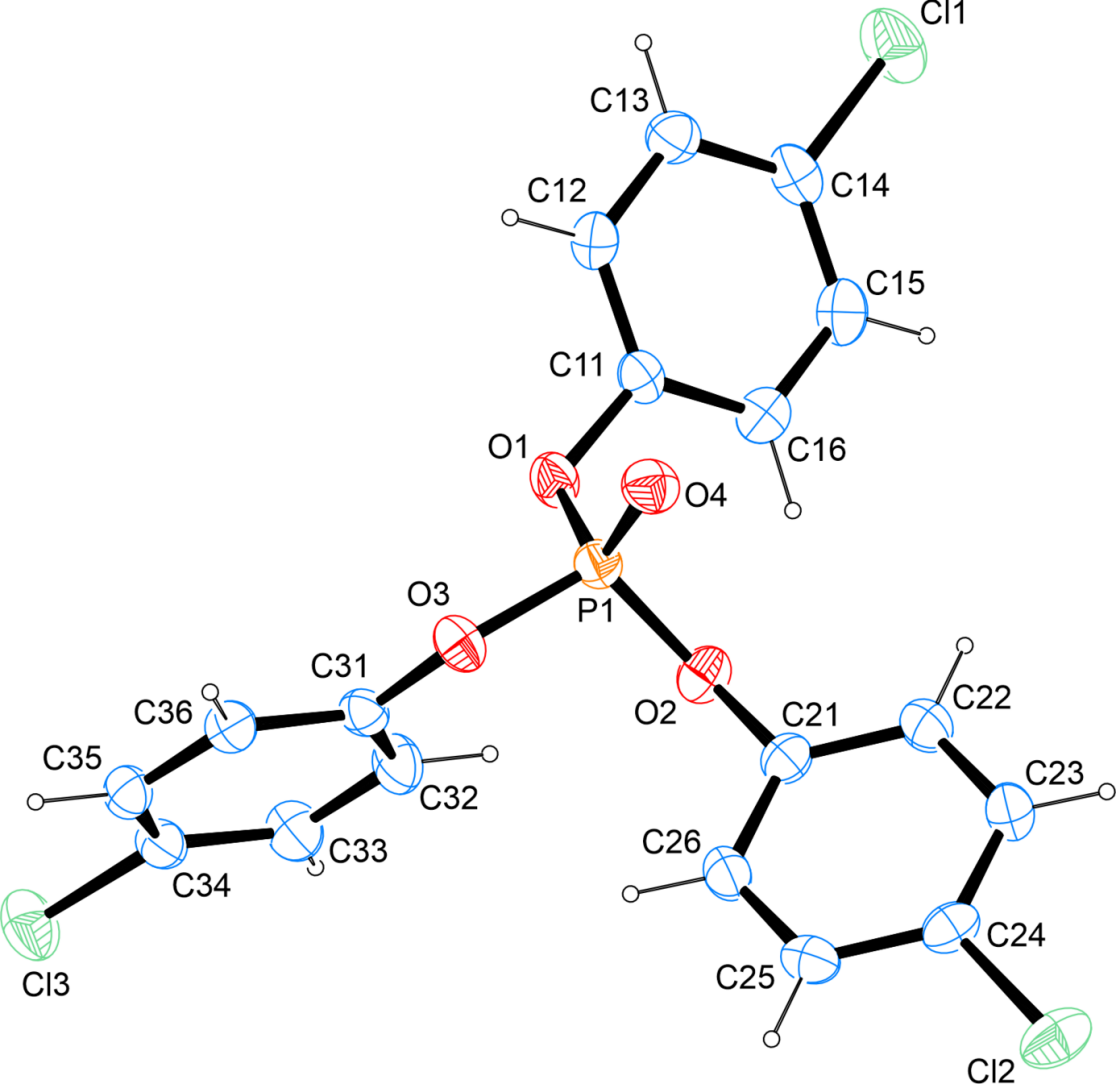
The mol­ecular structure of the title compound with displacement ellipsoids drawn at the 50% probability level.

The structure solution shows the presence of the title compound, C_18_H_12_Cl_3_O_4_P, a symmetric ester of phospho­ric acid derived from three equivalents of *para*-chloro­phenol (Fig. 1 ▸). The P—O bond lengths to the three aromatic alcohol moieties cover the range 1.5678 (13)–1.5806 (14) Å while the (formal) P=O double bond is found at a value of 1.4513 (14) Å. The carbon–chlorine bonds lie between 1.7388 (18) and 1.7422 (19) Å and are, therefore, in good agreement with other aromatic C—Cl bonds whose metrical parameters have been deposited with the Cambridge Structural Database (Groom *et al.*, 2002 ▸). The O—P—O angles span the range 101.50 (7)–118.37 (8)° with the largest three angles all involving the lone oxygen atom. The least-squares planes as defined by the respective carbon atoms of the C11, C21 and C31 aromatic rings enclose angles of 49.42 (8) (C11/C21), 69.83 (8) (C11/C31) and 77.48 (9)° (C21/C31). In comparison to the thio­nated analogue of the title compound (Hernandez *et al.*, 2006 ▸), the P—O bonds as well as the C—Cl bonds are all found at slightly larger values in the sulfur-derivative.

**Figure 2:**
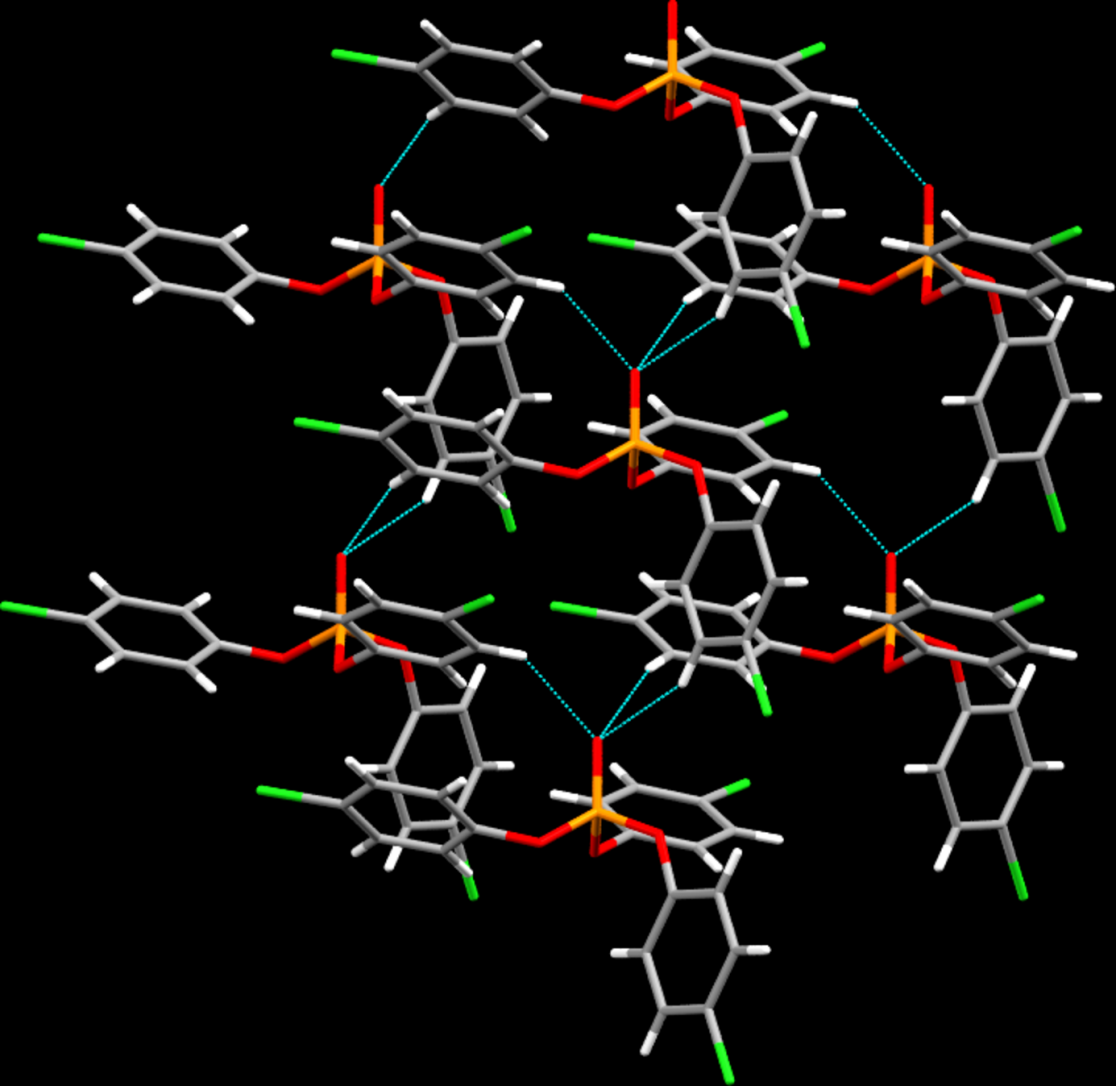
Inter­molecular contacts viewed approximately along [010].

In the crystal of C_18_H_12_Cl_3_O_4_P, C—H⋯O contacts shorter than 0.1 Å less than the sum of the van der Waals radii are apparent. The latter are supported by one of the hydrogen atoms in the *ortho*-position to the chlorine atom on each of the aromatic rings and uniformly employ the (formally) double bonded oxygen atom O4 as acceptor (Table 1 ▸). In terms of graph-set analysis (Etter *et al.*, 1990 ▸; Bernstein *et al.*, 1995 ▸), these contacts require a *
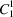
(7) 
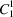
(7) 
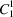
(7)* descriptor on the unary level. In total, the mol­ecules are connected into sheets lying perpendicular to the crystallographic *b* axis (Fig. 2 ▸). Two of the aromatic systems experience stabilization through π-stacking inter­actions with the shortest inter­centroid distance measured at 3.7544 (10) Å.

## Synthesis and crystallization

The title compound was isolated as an accidental hydrolysis by-product upon the synthesis of the symmetric *para*-chloro­phen­oxy­phospho­rane from PCl_5_ and *para*-chloro­phenol according to a published procedure (Ramirez *et al.*, 1968 ▸). A crystal suitable for the diffraction study was taken directly from the crystallized oily product.

## Refinement

Crystal data, data collection and structure refinement details are summarized in Table 2 ▸.

## Supplementary Material

Crystal structure: contains datablock(s) I. DOI: 10.1107/S2414314624010617/hb4490sup1.cif

Structure factors: contains datablock(s) I. DOI: 10.1107/S2414314624010617/hb4490Isup2.hkl

Supporting information file. DOI: 10.1107/S2414314624010617/hb4490Isup3.cml

CCDC reference: 2396855

Additional supporting information:  crystallographic information; 3D view; checkCIF report

## Figures and Tables

**Table 1:** Hydrogen-bond geometry (Å, °)

*D*—H⋯*A*	*D*—H	H⋯*A*	*D*⋯*A*	*D*—H⋯*A*
C15—H15⋯O4^i^	0.95	2.42	3.324 (2)	159
C25—H25⋯O4^ii^	0.95	2.40	3.176 (2)	139
C33—H33⋯O4^iii^	0.95	2.59	3.423 (2)	147

Symmetry codes: (i) 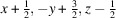; (ii) 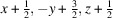; (iii) 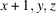.

**Table 2:** Experimental details

Crystal data	
Chemical formula	C_18_H_12_Cl_3_O_4_P
*M*_r_	429.60
Crystal system, space group	Monoclinic, *P*2_1_/*n*
Temperature (K)	200
*a*, *b*, *c* (Å)	7.5771 (5), 21.1280 (14), 11.2665 (7)
β (°)	95.835 (2)
*V* (Å^3^)	1794.3 (2)
*Z*	4
Radiation type	Mo *K*α
μ (mm^−1^)	0.62
Crystal size (mm)	0.60 × 0.14 × 0.13

Data collection	
Diffractometer	Bruker APEXII CCD
Absorption correction	Multi-scan (*SADABS*; Krause *et al.*, 2015 ▸)
*T*_min_, *T*_max_	0.792, 1.000
No. of measured, independent and observed [*I* > 2σ(*I*)] reflections	58447, 4427, 3920
*R*_int_	0.036
(sin θ/λ)_max_ (Å^−1^)	0.667

Refinement	
*R*[*F*^2^ > 2σ(*F*^2^)], *wR*(*F*^2^), *S*	0.038, 0.097, 1.12
No. of reflections	4427
No. of parameters	235
H-atom treatment	H-atom parameters constrained
Δρ_max_, Δρ_min_ (e Å^−3^)	0.37, −0.44

Computer programs: *APEX2* and *SAINT* (Bruker, 2016 ▸), *SHELXS97* (Sheldrick 2008 ▸), *ORTEP-3 for Windows* (Farrugia, 2012 ▸), *Mercury* (Macrae *et al.*, 2020 ▸), *SHELXL2019/3* (Sheldrick, 2015 ▸) and *PLATON* (Spek, 2020 ▸).

## full crystallographic data

### Tris(4-chlorophenyl) phosphate Crystal data

**Table:**

C_18_H_12_Cl_3_O_4_P	*F*(000) = 872
*M**_r_* = 429.60	*D*_x_ = 1.590 Mg m^−^^3^
Monoclinic, *P*2_1_/*n*	Mo *K*α radiation, λ = 0.71073 Å
*a* = 7.5771 (5) Å	Cell parameters from 9220 reflections
*b* = 21.1280 (14) Å	θ = 2.7–28.3°
*c* = 11.2665 (7) Å	µ = 0.62 mm^−^^1^
β = 95.835 (2)°	*T* = 200 K
*V* = 1794.3 (2) Å^3^	Rod, colourless
*Z* = 4	0.60 × 0.14 × 0.13 mm

### Tris(4-chlorophenyl) phosphate Data collection

**Table:**

Bruker APEXII CCD diffractometer	3920 reflections with *I* > 2σ(*I*)
φ and ω scans	*R*_int_ = 0.036
Absorption correction: multi-scan (SADABS; Krause *et al.*, 2015)	θ_max_ = 28.3°, θ_min_ = 2.1°
*T*_min_ = 0.792, *T*_max_ = 1.000	*h* = −10→9
58447 measured reflections	*k* = −28→28
4427 independent reflections	*l* = −15→15

### Tris(4-chlorophenyl) phosphate Refinement

**Table:**

Refinement on *F*^2^	Primary atom site location: structure-invariant direct methods
Least-squares matrix: full	Secondary atom site location: difference Fourier map
*R*[*F*^2^ > 2σ(*F*^2^)] = 0.038	Hydrogen site location: inferred from neighbouring sites
*wR*(*F*^2^) = 0.097	H-atom parameters constrained
*S* = 1.12	*w* = 1/[σ^2^(*F*_o_^2^) + (0.0344*P*)^2^ + 1.6089*P*] where *P* = (*F*_o_^2^ + 2*F*_c_^2^)/3
4427 reflections	(Δ/σ)_max_ = 0.001
235 parameters	Δρ_max_ = 0.37 e Å^−^^3^
0 restraints	Δρ_min_ = −0.43 e Å^−^^3^

### Tris(4-chlorophenyl) phosphate Special details

**Table:**

Geometry. All esds (except the esd in the dihedral angle between two l.s. planes) are estimated using the full covariance matrix. The cell esds are taken into account individually in the estimation of esds in distances, angles and torsion angles; correlations between esds in cell parameters are only used when they are defined by crystal symmetry. An approximate (isotropic) treatment of cell esds is used for estimating esds involving l.s. planes.
Refinement. The carbon-bound H atoms were placed in calculated positions (C—H = 0.95 Å) and were included in the refinement in the riding model approximation, with *U*_iso_(H) set to 1.2*U*_eq_(C).

### Tris(4-chlorophenyl) phosphate Fractional atomic coordinates and isotropic or equivalent isotropic displacement parameters (Å^2^)

**Table:**

	*x*	*y*	*z*	*U*_iso_*/*U*_eq_	
Cl1	0.17450 (8)	0.67130 (3)	0.25722 (4)	0.04555 (15)	
Cl2	0.15619 (7)	0.96171 (2)	1.13729 (5)	0.04071 (14)	
Cl3	0.96260 (7)	0.50899 (3)	1.16357 (5)	0.03942 (14)	
P1	0.23268 (6)	0.67194 (2)	0.87255 (4)	0.02265 (11)	
O1	0.31794 (18)	0.63308 (6)	0.77432 (11)	0.0272 (3)	
O2	0.34438 (17)	0.73521 (6)	0.87549 (12)	0.0277 (3)	
O3	0.29349 (17)	0.63395 (6)	0.98911 (11)	0.0269 (3)	
O4	0.04172 (17)	0.68072 (6)	0.85985 (12)	0.0298 (3)	
C11	0.2781 (2)	0.64485 (8)	0.65109 (15)	0.0227 (3)	
C12	0.1892 (2)	0.59829 (8)	0.58403 (16)	0.0262 (4)	
H12	0.150962	0.560988	0.620952	0.031*	
C13	0.1561 (2)	0.60655 (9)	0.46160 (17)	0.0280 (4)	
H13	0.094852	0.575032	0.413415	0.034*	
C14	0.2138 (2)	0.66143 (9)	0.41085 (16)	0.0276 (4)	
C15	0.3042 (3)	0.70793 (9)	0.47845 (18)	0.0298 (4)	
H15	0.343241	0.745139	0.441633	0.036*	
C16	0.3373 (2)	0.69956 (9)	0.60092 (17)	0.0273 (4)	
H16	0.399366	0.730840	0.649255	0.033*	
C21	0.2917 (2)	0.78820 (8)	0.93942 (16)	0.0237 (3)	
C22	0.2113 (3)	0.83806 (9)	0.87600 (16)	0.0288 (4)	
H22	0.186261	0.835618	0.791773	0.035*	
C23	0.1678 (3)	0.89184 (9)	0.93736 (17)	0.0292 (4)	
H23	0.112444	0.926854	0.895784	0.035*	
C24	0.2060 (2)	0.89370 (9)	1.05993 (17)	0.0269 (4)	
C25	0.2854 (2)	0.84366 (9)	1.12328 (16)	0.0279 (4)	
H25	0.309330	0.845798	1.207611	0.033*	
C26	0.3298 (2)	0.79007 (9)	1.06137 (16)	0.0264 (4)	
H26	0.385733	0.755121	1.102768	0.032*	
C31	0.4588 (2)	0.60700 (8)	1.02654 (15)	0.0229 (3)	
C32	0.6164 (3)	0.62701 (9)	0.98716 (17)	0.0300 (4)	
H32	0.617447	0.660803	0.931603	0.036*	
C33	0.7737 (3)	0.59701 (10)	1.03000 (18)	0.0315 (4)	
H33	0.883513	0.610130	1.004259	0.038*	
C34	0.7679 (2)	0.54797 (9)	1.11032 (16)	0.0260 (4)	
C35	0.6100 (2)	0.52804 (8)	1.14972 (15)	0.0256 (4)	
H35	0.608753	0.494033	1.204801	0.031*	
C36	0.4536 (2)	0.55828 (8)	1.10788 (15)	0.0244 (3)	
H36	0.344168	0.545645	1.134817	0.029*	

### Tris(4-chlorophenyl) phosphate Atomic displacement parameters (Å^2^)

**Table:**

	*U*^11^	*U*^22^	*U*^33^	*U*^12^	*U*^13^	*U*^23^
Cl1	0.0594 (4)	0.0555 (3)	0.0228 (2)	0.0116 (3)	0.0091 (2)	0.0070 (2)
Cl2	0.0412 (3)	0.0326 (2)	0.0492 (3)	−0.0024 (2)	0.0087 (2)	−0.0158 (2)
Cl3	0.0279 (2)	0.0446 (3)	0.0451 (3)	0.0055 (2)	0.0005 (2)	0.0124 (2)
P1	0.0239 (2)	0.0231 (2)	0.0209 (2)	0.00231 (16)	0.00230 (16)	−0.00020 (16)
O1	0.0338 (7)	0.0262 (6)	0.0214 (6)	0.0082 (5)	0.0025 (5)	0.0001 (5)
O2	0.0298 (7)	0.0248 (6)	0.0298 (6)	−0.0003 (5)	0.0088 (5)	−0.0018 (5)
O3	0.0248 (6)	0.0327 (7)	0.0231 (6)	0.0027 (5)	0.0031 (5)	0.0053 (5)
O4	0.0249 (6)	0.0321 (7)	0.0319 (7)	0.0031 (5)	0.0009 (5)	−0.0025 (5)
C11	0.0233 (8)	0.0246 (8)	0.0206 (8)	0.0071 (6)	0.0044 (6)	0.0004 (6)
C12	0.0290 (9)	0.0227 (8)	0.0274 (9)	0.0032 (7)	0.0058 (7)	0.0025 (7)
C13	0.0286 (9)	0.0284 (9)	0.0269 (9)	0.0032 (7)	0.0021 (7)	−0.0036 (7)
C14	0.0285 (9)	0.0350 (9)	0.0205 (8)	0.0103 (7)	0.0076 (7)	0.0038 (7)
C15	0.0295 (9)	0.0283 (9)	0.0334 (9)	0.0033 (7)	0.0117 (8)	0.0064 (7)
C16	0.0247 (8)	0.0264 (9)	0.0316 (9)	−0.0002 (7)	0.0065 (7)	−0.0013 (7)
C21	0.0222 (8)	0.0228 (8)	0.0265 (8)	−0.0021 (6)	0.0048 (6)	−0.0010 (6)
C22	0.0338 (10)	0.0303 (9)	0.0218 (8)	0.0008 (7)	0.0010 (7)	0.0019 (7)
C23	0.0310 (9)	0.0268 (9)	0.0293 (9)	0.0036 (7)	0.0010 (7)	0.0046 (7)
C24	0.0239 (8)	0.0254 (8)	0.0321 (9)	−0.0040 (7)	0.0064 (7)	−0.0056 (7)
C25	0.0262 (9)	0.0350 (10)	0.0219 (8)	−0.0045 (7)	0.0007 (7)	−0.0002 (7)
C26	0.0253 (8)	0.0272 (8)	0.0261 (8)	0.0004 (7)	0.0004 (7)	0.0059 (7)
C31	0.0253 (8)	0.0252 (8)	0.0182 (7)	0.0012 (6)	0.0018 (6)	−0.0008 (6)
C32	0.0286 (9)	0.0308 (9)	0.0306 (9)	−0.0035 (7)	0.0034 (7)	0.0104 (7)
C33	0.0256 (9)	0.0361 (10)	0.0330 (10)	−0.0044 (7)	0.0046 (7)	0.0070 (8)
C34	0.0252 (8)	0.0277 (9)	0.0243 (8)	0.0013 (7)	−0.0010 (7)	0.0005 (7)
C35	0.0310 (9)	0.0247 (8)	0.0213 (8)	−0.0011 (7)	0.0045 (7)	0.0032 (6)
C36	0.0263 (8)	0.0265 (8)	0.0213 (8)	−0.0018 (7)	0.0064 (6)	0.0009 (6)

### Tris(4-chlorophenyl) phosphate Geometric parameters (Å, º)

**Table:**

Cl1—C14	1.7388 (18)	C21—C26	1.376 (3)
Cl2—C24	1.7418 (19)	C21—C22	1.379 (3)
Cl3—C34	1.7422 (19)	C22—C23	1.387 (3)
P1—O4	1.4513 (14)	C22—H22	0.9500
P1—O3	1.5678 (13)	C23—C24	1.382 (3)
P1—O1	1.5690 (13)	C23—H23	0.9500
P1—O2	1.5806 (14)	C24—C25	1.379 (3)
O1—C11	1.413 (2)	C25—C26	1.389 (3)
O2—C21	1.411 (2)	C25—H25	0.9500
O3—C31	1.401 (2)	C26—H26	0.9500
C11—C12	1.374 (3)	C31—C36	1.381 (2)
C11—C16	1.381 (3)	C31—C32	1.382 (3)
C12—C13	1.388 (3)	C32—C33	1.392 (3)
C12—H12	0.9500	C32—H32	0.9500
C13—C14	1.383 (3)	C33—C34	1.379 (3)
C13—H13	0.9500	C33—H33	0.9500
C14—C15	1.381 (3)	C34—C35	1.383 (3)
C15—C16	1.388 (3)	C35—C36	1.386 (3)
C15—H15	0.9500	C35—H35	0.9500
C16—H16	0.9500	C36—H36	0.9500
			
O4—P1—O3	110.68 (8)	C23—C22—H22	120.6
O4—P1—O1	118.37 (8)	C24—C23—C22	119.00 (17)
O3—P1—O1	102.47 (7)	C24—C23—H23	120.5
O4—P1—O2	114.86 (8)	C22—C23—H23	120.5
O3—P1—O2	107.72 (7)	C25—C24—C23	122.09 (17)
O1—P1—O2	101.50 (7)	C25—C24—Cl2	118.61 (15)
C11—O1—P1	122.62 (11)	C23—C24—Cl2	119.29 (15)
C21—O2—P1	120.20 (11)	C24—C25—C26	118.69 (17)
C31—O3—P1	129.62 (11)	C24—C25—H25	120.7
C12—C11—C16	122.37 (16)	C26—C25—H25	120.7
C12—C11—O1	117.26 (15)	C21—C26—C25	119.23 (17)
C16—C11—O1	120.24 (16)	C21—C26—H26	120.4
C11—C12—C13	119.02 (17)	C25—C26—H26	120.4
C11—C12—H12	120.5	C36—C31—C32	121.64 (17)
C13—C12—H12	120.5	C36—C31—O3	114.70 (15)
C14—C13—C12	118.92 (17)	C32—C31—O3	123.66 (16)
C14—C13—H13	120.5	C31—C32—C33	119.12 (17)
C12—C13—H13	120.5	C31—C32—H32	120.4
C15—C14—C13	121.88 (17)	C33—C32—H32	120.4
C15—C14—Cl1	119.26 (15)	C34—C33—C32	119.09 (17)
C13—C14—Cl1	118.85 (15)	C34—C33—H33	120.5
C14—C15—C16	119.12 (17)	C32—C33—H33	120.5
C14—C15—H15	120.4	C33—C34—C35	121.70 (17)
C16—C15—H15	120.4	C33—C34—Cl3	119.91 (15)
C11—C16—C15	118.68 (17)	C35—C34—Cl3	118.38 (14)
C11—C16—H16	120.7	C34—C35—C36	119.20 (16)
C15—C16—H16	120.7	C34—C35—H35	120.4
C26—C21—C22	122.15 (17)	C36—C35—H35	120.4
C26—C21—O2	119.43 (16)	C31—C36—C35	119.24 (16)
C22—C21—O2	118.33 (16)	C31—C36—H36	120.4
C21—C22—C23	118.83 (17)	C35—C36—H36	120.4
C21—C22—H22	120.6		
			
O4—P1—O1—C11	48.94 (16)	C26—C21—C22—C23	0.1 (3)
O3—P1—O1—C11	170.98 (13)	O2—C21—C22—C23	−176.51 (16)
O2—P1—O1—C11	−77.74 (14)	C21—C22—C23—C24	−0.1 (3)
O4—P1—O2—C21	40.80 (15)	C22—C23—C24—C25	−0.3 (3)
O3—P1—O2—C21	−83.02 (14)	C22—C23—C24—Cl2	178.60 (15)
O1—P1—O2—C21	169.75 (13)	C23—C24—C25—C26	0.7 (3)
O4—P1—O3—C31	169.27 (14)	Cl2—C24—C25—C26	−178.23 (14)
O1—P1—O3—C31	42.15 (16)	C22—C21—C26—C25	0.3 (3)
O2—P1—O3—C31	−64.41 (16)	O2—C21—C26—C25	176.86 (16)
P1—O1—C11—C12	−113.75 (16)	C24—C25—C26—C21	−0.7 (3)
P1—O1—C11—C16	70.2 (2)	P1—O3—C31—C36	−157.17 (13)
C16—C11—C12—C13	−0.5 (3)	P1—O3—C31—C32	23.3 (3)
O1—C11—C12—C13	−176.52 (15)	C36—C31—C32—C33	0.4 (3)
C11—C12—C13—C14	0.0 (3)	O3—C31—C32—C33	179.97 (17)
C12—C13—C14—C15	0.4 (3)	C31—C32—C33—C34	0.2 (3)
C12—C13—C14—Cl1	179.27 (14)	C32—C33—C34—C35	−0.3 (3)
C13—C14—C15—C16	−0.4 (3)	C32—C33—C34—Cl3	179.11 (15)
Cl1—C14—C15—C16	−179.23 (14)	C33—C34—C35—C36	−0.3 (3)
C12—C11—C16—C15	0.6 (3)	Cl3—C34—C35—C36	−179.68 (14)
O1—C11—C16—C15	176.44 (15)	C32—C31—C36—C35	−1.0 (3)
C14—C15—C16—C11	−0.1 (3)	O3—C31—C36—C35	179.43 (15)
P1—O2—C21—C26	78.94 (19)	C34—C35—C36—C31	0.9 (3)
P1—O2—C21—C22	−104.39 (17)		

### Tris(4-chlorophenyl) phosphate Hydrogen-bond geometry (Å, º)

**Table:**

*D*—H···*A*	*D*—H	H···*A*	*D*···*A*	*D*—H···*A*
C15—H15···O4^i^	0.95	2.42	3.324 (2)	159
C25—H25···O4^ii^	0.95	2.40	3.176 (2)	139
C33—H33···O4^iii^	0.95	2.59	3.423 (2)	147

Symmetry codes: (i) *x*+1/2, −*y*+3/2, *z*−1/2; (ii) *x*+1/2, −*y*+3/2, *z*+1/2; (iii) *x*+1, *y*, *z*.
